# Measuring personal characteristics in applicants to German medical schools: Piloting an online Situational Judgement Test with an open-ended response format

**DOI:** 10.3205/zma001685

**Published:** 2024-06-17

**Authors:** Mirjana Knorr, Ina Mielke, Dorothee Amelung, Mahla Safari, Oana R. Gröne, Simon M. Breil, Alexander MacIntosh

**Affiliations:** 1 University Medical Center Hamburg-Eppendorf, Arbeitsgruppe Auswahlverfahren, Hamburg, Germany; 2 University of Heidelberg, Heidelberg, Germany; 3 University of Münster, Münster, Germany; 4 Acuity Insights, Toronto, Canada

**Keywords:** admission, situational judgement test, personal characteristics, Casper

## Abstract

**Objectives:**

Situational Judgement Tests (SJT) are a cost-efficient method for the assessment of personal characteristics (e.g., empathy, professionalism, ethical thinking) in medical school admission. Recently, complex open-ended response format SJTs have become more feasible to conduct. However, research on their applicability to a German context is missing. This pilot study tests the acceptability, reliability, subgroup differences, and validity of an online SJT with open-ended response format developed in Canada (“Casper”).

**Methods:**

German medical school applicants and students from Hamburg were invited to take Casper in 2020 and 2021. The test consisted of 12 video- and text-based scenarios, each followed by three open-ended questions. Participants subsequently evaluated their test experience in an online survey. Data on sociodemographic characteristics, other admission criteria (Abitur, TMS, HAM-Nat, HAM-SJT) and study success (OSCE) was available in a central research database (stav).

**Results:**

The full sample consisted of 582 participants. Test-takers’ global perception of Casper was positive. Internal consistency was satisfactory in both years (*α*=0.73; 0.82) while interrater agreement was moderate (ICC(1,2)=0.54). Participants who were female (*d*=0.37) or did not have a migration background (*d*=0.40) received higher scores. Casper scores correlated with HAM-SJT (*r*=.18) but not with OSCE communication stations performance. The test was also related to Abitur grades (*r*=-.15), the TMS (*r*=.18), and HAM-Nat logical reasoning scores (*r*=.23).

**Conclusion:**

This study provides positive evidence for the acceptability, internal consistency, and convergent validity of Casper. The selection and training of raters as well as the scenario content require further observation and adjustments to a German context to improve interrater reliability and predictive validity.

## Zusammenfassung

**Ziele:**

Situational Judgement Tests (SJTs) sind eine kosteneffiziente Methode zur Beurteilung von persönlichen Eigenschaften (z.B. Empathie, Professionalität, ethisches Denken) bei der Zulassung zum Medizinstudium. Die Durchführung komplexer SJTs mit offenem Antwortformat ist in jüngster Zeit einfacher geworden. Es fehlen jedoch Untersuchungen zu ihrer Anwendbarkeit im deutschen Kontext. Diese Pilotstudie testet die Akzeptanz, Reliabilität, Subgruppenunterschiede und Validität eines in Kanada entwickelten Online-SJTs mit offenem Antwortformat („Casper“).

**Methoden:**

Studienbewerber*innen aus Deutschland und Medizinstudierende aus Hamburg wurden eingeladen, Casper in den Jahren 2020 und 2021 zu absolvieren. Der Test bestand aus 12 video- und textbasierten Szenarien, auf die jeweils drei offene Fragen folgten. Die Teilnehmenden bewerteten anschließend ihre Testerfahrung in einer Online-Umfrage. Daten zu soziodemografischen Merkmalen, weiteren Zulassungskriterien (Abitur, TMS, HAM-Nat, HAM-SJT) und zum Studienerfolg (OSCE) waren in einer zentralen Forschungsdatenbank (stav) verfügbar.

**Ergebnisse:**

Die Gesamtstichprobe bestand aus 582 Teilnehmenden. Die allgemeine Wahrnehmung von Casper durch die Testteilnehmenden war positiv. Die interne Konsistenz war in beiden Jahren zufriedenstellend (*α*=0,73; 0,82), während die Interrater-Übereinstimmung mäßig war (*ICC*(1,2)=0,54). Weibliche Teilnehmerinnen (*d*=0,37) oder Teilnehmende ohne Migrationshintergrund (*d*=0,40) erzielten höhere Testwerte. Die Casper Testwerte korrelierten mit dem HAM-SJT (*r*=.18), aber nicht mit der Leistung in OSCE-Kommunikationsstationen. Der Test zeigte auch Zusammenhänge mit der Abiturnote (*r*=-.15), dem TMS (*r*=.18) und dem HAM-Nat-Subtest für logisches Denken (*r*=.23).

**Schlussfolgerungen:**

Die Studie liefert positive Belege für die Akzeptanz, interne Konsistenz und konvergente Validität von Casper. Die Auswahl und Schulung der Beurteiler*innen sowie die Inhalte der Szenarien bedürfen weiterer Untersuchungen und Anpassungen an den deutschen Kontext, um die Interrater-Reliabilität und prädiktive Validität zu verbessern.

## 1. Introduction

### 1.1. Background

Personal characteristics of future physicians such as ethical thinking, professionalism, and social skills, have gained increased importance in competency frameworks for medical education [1], [2], [3]. Likewise, these characteristics were emphasized in the “Masterplan Medizinstudium 2020”, a 2017 resolution by the federal and regional governments of Germany to regulate the reformation of medical curricula [4]. One of the directives in the resolution was to not exclusively focus on high-school grades or results of aptitude tests [5] but to attach more importance to personal characteristics in the admission process [4]. The current main methods used to evaluate such characteristics are traditional or multiple mini-interviews (MMIs) [6] and professional pre-qualifications (i.e. completed vocational training, volunteer work). However, both methods have limitations. Interviews are considered inefficient and resource-intensive for the assessment of an entire pool of multiple thousand applicants, especially considering the amount of interviewer time needed [7]. Although preliminary supporting evidence exists that (when controlling for Abitur grade and cognitive test performance) a vocational training can predict study success [8] it is yet unclear to what extent professional pre-qualifications are indicative of personal characteristics or clinical skills [9]. The fairness of professional pre-qualifications as selection criteria can also be questioned as not every applicant has the opportunity to volunteer or to complete a three-year vocational training.

Therefore, we suggest Situational Judgement Tests [10] as promising cost-efficient and evidence-based alternatives to interviews and professional pre-qualifications. SJTs present candidates with several short situation descriptions (scenarios) in a text or video format followed by instructions to identify what one would or should do in the described situation. Internationally, SJTs used for medical selection demonstrate good psychometric properties [11] with a recent meta-analysis reporting a pooled estimate of *r*=.32 for predicting interpersonal performance evaluations [12]. Traditionally, SJTs use a closed-ended response format (i.e., choosing from, rating, or ranking a list of response alternatives). Due to technological advances, open-ended response format SJTs (i.e., applicants provide their response to an SJT scenario in a written text or in an audio/video format) have recently become more feasible [13]. Research indicates that these types of response formats might reduce minority-majority differences (i.e., performance differences between natives and immigrants) because multiple choice formats require more cognitive resources to understand and compare each of the provided response options whereas open-ended questions can be responded to when the core dilemma of a scenario is understood [13]. In addition, it is assumed that open-ended response formats are less prone to faking [14]. In health-care selection, research on open-ended response format SJTs has focused on Casper (formerly known as: Computer-based Assessment for Sampling Personal Characteristics), a digitally administered SJT which is currently offered in English and French. In these studies, Casper demonstrated good acceptability and reliability [15], [16], fewer minority-majority performance differences compared to cognitive tests [17], and a correlation with later performance at licensure exam subtests which focus on communicational and ethical aspects [18]. 

Despite their potential benefits compared to interviews or professional pre-qualifications, SJTs currently play a minor role in German medical admission and supporting evidence is limited. The University of Heidelberg developed a video-based SJT for self-assessment purposes [19] and the University of Hamburg recently introduced a paper-pencil SJT (Hamburger Situational Judgement Test, HAM-SJT) for their undergraduate admission process [20]. Both SJTs use a closed-ended response format and to our knowledge, an SJT with an open-ended format has not yet been tested in a medical selection process in Germany.

### 1.2. Aim of the study

In this study, we piloted Casper as an online-SJT with an open-ended response format that could potentially be administered for high-stakes testing in Germany in the future. Our goal was to analyze the acceptability, reliability, subgroup performance differences as well as the convergent (i.e., relationship to other measures of personal characteristics) and discriminant (i.e., relationship to cognitive admission criteria) validity in comparison to the international evidence on Casper.

## 2. Methods

### 2.1. Procedure

This study took place on five test dates over the summers of 2020 and 2021. Applicants were invited to sign up for one of the test dates if they had registered for any of the major German medical school admission tests (Test für medizinische Studiengänge (TMS), Hamburger Naturwissenschaftstest (HAM-Nat), Hamburger Situational Judgement Test (HAM-SJT), see table 1 (Tab. 1)) and had indicated their interest to participate in research studies on student selection. In addition, all medical students at the University of Hamburg, irrespective of study year, received an invitation to take part in this study via an electronic student newsletter. To incentivize study participation, all participants received feedback on their Casper performance and had the chance to win vouchers over 50€ for an online store. Test fees were not charged in this study but can roughly be estimated to range between 46 and 95 EUR based on the current pricing (2024) in North-America. 

### 2.2. Casper

Casper focuses on assessing inter-individual differences in ten personal characteristics including collaboration, communication, empathy, equity, ethics, motivation, problem solving, professionalism, resilience, and self-awareness. Each scenario is usually designed to measure more than one characteristic and for each participant the composition of different scenarios ensures all ten characteristics are covered. In line with findings that such characteristics cannot be reliably discriminated within SJTs [21], [22], Casper only provides one overall score. 

In this study, the assessment consisted of eight video and four text scenarios. Each scenario was accompanied by three questions and participants were asked to provide their responses in an open text format within a 5-minute time limit per scenario. English language scenarios were selected from an existing pool of which six were used both in 2020 and 2021 while the six other scenarios varied between years to include a broader variety of scenarios. Video dialogues and questions were translated into German by the German research team: A linguist and public health scientist fluent in English wrote the transcripts of the video dialogues, which then were translated into German by a German-native psychologist. This translation was reviewed by a third person (German-native psychologist). Discrepancies were discussed and solved within the team. Videos were either subtitled (2020) or provided with a voice-over (2021). Participants took the test via the Casper online-platform. English language examples of typical Casper scenarios and questions can be found on the official website [https://acuityinsights.app/test-prep-casper/].

In 2020, 52 faculty staff members and student assistants from different German universities rated participants’ responses. Of these, 15 provided their ratings again the following year. In line with widening participation policies it is recommended to include raters that reflect patient diversity and promote inclusivity in medicine within rater-based selection tools [23], [24], [25] in order to reduce bias and enhance fairness by considering different perspectives and backgrounds in the evaluation process. Thus, to diversify the rater pool for the 2021 study, we recruited 11 additional community raters via online platforms for temporary job offers and e-mail lists of associations for people with a migration background. All raters completed an online on-demand training offered in English (2020) or German (2021) language. On average, raters needed 46.19 seconds (*SD*=22.72) for the rating of one response with a mean count of 125.60 words (*SD*=38.05). Faculty raters completed their ratings within their working hours while community raters were compensated with a voucher for an online store (0.50 EUR per rated response). After completing their ratings, raters in the 2021 study were asked to provide sociodemographic data in a voluntary survey.

Each response to a scenario was evaluated by one (2020) or two raters (2021) on a 9-point global rating scale ranging from 1=“poor” to 9=“excellent” with no specific behavioral anchors. For each scenario, raters received guidelines on how to consider the specific construct(s) the scenario was designed to measure in their ratings. They were instructed to rate the quality of each response relative to the corresponding ones provided by other participants. 

Raters were assigned responses through an online rating platform. After a certain number of ratings, they were able to switch to a new scenario to avoid fatigue. For each individual candidate, an algorithm of the online platform ensured that each scenario was rated by a different rater. In case of two raters, both ratings were averaged to generate a scenario score. The overall Casper score is delivered as a mean over twelve scenarios z-standardized within a cohort.

### 2.3. Other measures

All study participants had previously agreed to take part in an ongoing research project (Studierendenauswahlverbund, “stav”, [https://www.projekt-stav.de/index.php] where admission data, study performance data of admitted students, and data from other research studies and a sociodemographic questionnaire (see attachment 1 ) are matched and stored in a central database. Casper data could thereby be matched to the following data sources available in this database. A summary of all instruments can also be found in table 1 (Tab. 1). 

#### 2.3.1. Acceptability

Upon completion of the Casper test, participants were directed to an online survey about their test experience. In addition to an overall evaluation of Casper on a 10-point scale, candidates were asked, for example, to indicate their perception of the fairness and difficulty of Casper on 7-point scales (the higher the evaluation, the more favorable; see attachment 2 ). Survey data was only available for the 2020 test dates.

#### 2.3.2. Sociodemographic characteristics

To compare this study to previous findings on subgroup differences in SJTs [17], [26], [27], we included gender, parents’ highest level of education (i.e., at least one of the parents holds an academic degree) as indicator for socio-economic status (SES) as well as “migration background” as indicator for ethnicity/nationality. Following the definition of the German census [28] a migration background was considered if at least one of the following was true: the person was not born in Germany, has a non-German citizenship, or one of the parents was not born in Germany.

#### 2.3.2. Validity

To study convergent validity, two additional measures were included: the *HAM-SJT* and communication performance in an Objective Structured Clinical Exam (*OSCE*). The *HAM-SJT* is a paper-pencil SJT with a closed-ended response format that was added to the admissions process to medical school at the University of Hamburg in 2020 [20], [29]. Students at the University of Hamburg typically take their first OSCE, an exam that consists of several short standardized interactions (stations) evaluated by raters [30], after one and a half years of studies. Since medical students from all cohorts were invited to take part in this study, our participants took this OSCE between 2016 and 2022. Between these years the twelve stations of this OSCE were comparable in terms of content and rating checklists. We used the results (in percent) of two stations with simulated patients designed to target communication skills (communication skills station, history taking station) [31]. Data for the communication skills station was only available for students who took the OSCE before the summer of 2020 because this station could not take place during the COVID-19 pandemic. 

For the analysis of discriminant validity we compared the Casper results to cognitive admission criteria including the German *Abitur grade* (equivalent to school-leaving grade point average), performance at the cognitive admission test *HAM-Nat*, a multiple-choice test with subtests on knowledge in natural sciences [32], arithmetic problem solving, and logical reasoning, and performance at the *Test für medizinische Studiengänge (TMS)*, a subject-specific admission test for medicine and other healthcare studies [33]. 

### 2.4. Data analysis

All analyses were conducted in R-4.2.1 [https://www.r-project.org/]. For the analysis of participants’ responses to the acceptability questionnaire, we calculated basic descriptive statistics for quantitative evaluations and counted the frequencies of commonly mentioned topics in open text format questions using MAXQDA 2022 [https://www.maxqda.com/de/]. Reliability of Casper was analyzed in terms of internal consistency over 12 scenarios (Cronbach’s alpha). For responses that were rated by two independent raters (2021 sample), we analyzed interrater agreement by means of intra class correlation (ICC(1,2)). We investigated individual subgroup differences in mean performance with Welch *t*-tests for independent samples; effect sizes were reported as Cohen’s *d*. Convergent and discriminant validity was analyzed using Pearson correlations. 

We based analyses of subgroup differences and validity on the overall sample. For cases in which participants took part in both years, the z-score of the more recent Casper date (2021) was used. Unpaired Welch *t*-Tests and Mann-Whitney-U-Tests were conducted to ensure that performance on study variables was comparable between study cohorts. The level of significance for all analyses was *α*=.05. The R code, a full data analysis report, all appendices, and information on how to request the original data can be found at [https://osf.io/9daz3/]. 

## 3. Results

### 3.1. Participants and raters

Overall, 582 individuals participated in this pilot study including 74 medical students and 508 applicants. Twenty participants took the Casper in both 2020 and 2021. Participants’ mean age was 21 years (*SD*=3.30). Further sociodemographic information was available for around 64% of the participants. In this subsample, 19% identified as male, 36% had a migration background, and 71% had at least one parent holding a university degree (see table 2 (Tab. 2)). Age, performance on Casper and other study variables were largely comparable between study cohorts (see attachment 3 , p.1-2). Only HAM-SJT performance was significantly better in the 2021 cohort compared to the 2020 cohort (*W*=3773.5, *p*<.001, *d*=0.62). Applicants and medical students did not differ in their average Casper performance (*t*(91.226)=-1.16, *p*=0.25, *d*=0.16). Average performance in six video scenarios that were used both in 2020 (subtitles) and 2021 (voice-over) did not differ between years (*t*(465.16)=-0.48, *p*=0.63, *d*=0.04).

Of the 26 raters in 2021, 15 of the faculty and 6 of the community raters provided demographic data (see table 3 (Tab. 3)). Most notably, community raters had a more diverse educational background as compared to faculty raters (33% vs. 83% holding a university degree).

### 3.2. Acceptability

Overall, participants of the 2020 study evaluated Casper favorably with a mean rating of 7.55 (*SD*=1.64, *n*=368) on a 10-point scale. On 7-point scales, participants indicated that they were satisfied with their overall test experience (*M*=5.40, *SD*=1.19, *n*=367) and perceived Casper as rather fair (*M*=5.24, *SD*=1.26, *n*=354). Participants evaluated Casper as a bit less stressful when asked to compare it to other exams in general (*M*=3.24, *SD*=1.50, *n*=359) and perceived it as neither difficult nor easy (*M*=4.08, *SD*=1.21, *n*=356). In the open text format questions, the most frequently criticized aspect regarding the test experience was the short response time which made some participants feel that the test could systematically disadvantage applicants with less typing experience *(n*=24) (see attachment 2 for full results).

### 3.3. Reliability

The internal consistency for Casper scenario scores was *α*=0.73, 95% CI [0.69, 0.77] in 2020 and *α*=0.82, 95% CI [0.79, 0.86] in 2021. For responses evaluated by two raters in 2021, overall interrater agreement was ICC(1,2)=0.54. Re-test reliability for twenty participants who completed Casper in both years was *ρ*=0.29 (Spearman’s rank correlation).

### 3.4. Subgroup differences

Single group comparisons revealed that female participants (*t*(107.16)=2.73, *p*=0.01, *d*=0.37) and participants without a migration background (*t*(263.09)=3.65, *p*<.001, *d*=0.40) showed a better mean Casper performance compared to male participants and participants with a migration background, respectively. Casper performance did not significantly differ depending on parents’ level of education (*t*(203.67)=1.30, *p*=0.19, *d*=0.15). Follow-up regression analyses with Casper performance as outcome variable revealed that adding native language as predictor explained the effect of migration background while gender and language remained significant predictors when controlling for cognitive criteria (see table 4 (Tab. 4)).

### 3.5. Convergent and discriminant validity

With respect to other measures of personal characteristics, Casper had a significant relationship with HAM-SJT performance (*r*=.18, *p*=.004, *n*=263) but was neither related to performance at the OSCE history taking station (*r*=-.09, *p*=.37, *n*=94) nor to the communication skills station (*r*=.08, *p*=.57, *n*=55). 

Regarding cognitive admission measures, Casper performance had significant correlations with the Abitur grade (*r*=-.15, *p*=.01, *n*=354; i.e. the better the Abitur grade, the better Casper performance), TMS performance (*r*=.18, *p*=.001, *n*=371), and the logical reasoning subtest of the HAM-Nat (*r*=.23, *p*<.001, *n*=270). On the other hand, it did not correlate with the HAM-Nat science (*r*=.04, *p*=.46, *n*=270) nor with the arithmetic problem solving subtest (*r*=.08, *p*=.18, *n*=270) (see table 1 (Tab. 1)). Attachment 3 includes a full correlation table for all study variables.

## 4. Discussion

In German medical education, text-based and video-based SJTs have been developed and suggested for the (self-)assessment, teaching and monitoring of relevant skills such as communication or professional behavior of medical school applicants and students [19], [20], [34], [35], [36]. While all these examples rely on a closed-ended response format, this is the first study piloting an online-SJT with open-ended response format in a German medical admission context. 

Similar to Canadian reports on Casper [16], participants’ perception of Casper was favorable and internal consistency was good. These results also align with positive perceptions as well as satisfactory internal consistency values for the Heidelberg video-SJT (0.81≤*α*≤.83) [19] and HAM-SJT (0.62≤*α*≤.82) [37]. On the other hand, interrater agreement in our study was only moderate and diverged from the high rater agreement (0.95) found in the Canadian pilot study of Casper [15]. In the small subsample of participants who sat the test twice, test-retest reliability was low. This might be explained by individual differences in participants’ personal development within the one-year time span between the two test applications but also by changes to the test format between both test applications (i.e. use of different scenarios, voice-over, inclusion of community raters). Nevertheless, the subsample in our study was too small (n=20) to draw definite conclusions and a follow-up study with a targeted test-retest design would be necessary.

Our study revealed significant performance differences in favor of females and participants without a migration background that are in line with a North-American study on Casper [17]. Our follow-up analyses suggest that native language rather than migration background was related to performance differences which diverges from findings in a U.S. study where differences in ethnicity remained when controlled for language use [38]. The open-ended response format did therefore not provide an advantage over the HAM-SJT which similarly showed performance differences depending on native language (*d*=0.24) [37] or the Heidelberg video-SJT which did not show any significant differences [19].

In support of the convergent and discriminant validity of the test, Casper performance was related to HAM-SJT performance but not to the HAM-Nat science and arithmetic problem solving subtests. Likewise, the Canadian Casper had not been found to be related to the MCAT science subtests [15]. On the other hand, we found weak correlations with the Abitur grade, TMS performance, and the HAM-Nat logical reasoning subtests. The weak reliability values of the HAM-Nat logical reasoning and arithmetic problem solving subtests might have affected the significance and magnitude of the correlation with Casper. Nevertheless, we found a similarly small significant correlation between TMS and Casper pointing in a similar direction and results are also in line with findings that Casper correlates with the verbal reasoning part of the MCAT [15]. This suggests that the cognitive but also non-cognitive competencies reflected in these measures (such as motivation, flexibility, or self-management in Abitur grades [39]) could be beneficial for Casper performance. The results also point to a somewhat higher cognitive load in Casper compared to the HAM-SJT or Heidelberg video-SJT which were either negatively related to Abitur grade, TMS and HAM-Nat or not at all [19], [20].

Finally, we did not find any relationship between Casper and two OSCE stations that address communication skills. Thus, we could not replicate positive evidence of predictive validity for the North-American Casper where Casper was related to MMI performance as well as to national licensure exams [15], [18]. HAM-SJT pilot studies, on the other hand, could demonstrate small but significant correlations with subsequent MMI (*r*=0.22) [20] and OSCE performance (*r*=0.20) [37].

### Limitations

We applied different measures of quality assurance during rater training and the rating process including repeated training rounds if statistics from test ratings fall below pre-determined benchmarks, or temporary retention of raters if they submit their ratings within less time than it needs to read a candidate’s response. However, in this pilot these measures were not employed to the same degree as they are in the high-stakes application of Casper. The moderate interrater agreement found in this study highlights the importance of continuously monitoring the rating process and providing feedback to raters.

In the 2021 study, we recruited additional community raters with the aim to diversify the rater pool. Although demographic data somewhat suggest that community raters differed from faculty raters in terms of their level of education, the lower participation rate of community raters in the follow-up survey (55%) makes it difficult to draw definite conclusions about the diversity of our rater pool. Future studies on rater-based selection tools would benefit from a systematic assessment and variation of raters’ sociodemographic characteristics to be able to explore how diverse rater backgrounds impact outcomes of high-stakes selection.

For this pilot, we used scenarios that were developed and previously tested in a North-American high-stakes context. However, it remains unclear whether any cultural differences related to scenario content had an impact on study results. In addition, the participants in our study were volunteers and their motivation to perform will differ from that in a high-stakes selection context. Lastly, we only invited applicants to this study who registered for the TMS and/or HAM-Nat and aimed at improving their chances of gaining a study place. Our sample is therefore not representative of the population of all those interested in studying medicine and likely excludes applicants with a high Abitur grade as well as those who are discouraged by the current selection system and do not apply. However, the latter group might potentially benefit from a non-cognitive test like Casper. For future assessments, it is advised to develop the test content in the culture and language where the test is administered and to confirm the psychometric properties within an actual selection procedure.

### Implications for practice and research

A recent study revealed that physicians and medical students in Hamburg do not represent the general population especially in terms of their socio-economic and ethnic background [40]. Medical schools that adopt a widening participation policy need to pay attention to how underrepresented groups perform on a selection criterion when compiling and weighting their selection criteria to minimize adverse impact. Participants’ performance in our study did not differ depending on socio-economic background. However, we could only use parents’ level of education as indicator. The use of additional indicators such as parents’ income or living conditions [40] in future studies might provide a more comprehensive picture. Although our results suggest a potential disadvantage for applicants whose first language is not German, it has been argued internationally that SJTs like Casper can mitigate the often more severe subgroup differences in cognitive tests and thereby potentially widen access to medical school [17], [27]. While preliminary data on the HAM-Nat suggests that applicants without a migration background perform better on the two reasoning subtests (0.24≤*d*≤0.32) and applicants with a higher socio-economic background perform better on all three HAM-Nat subtest (0.06≤*d*≤0.25), the magnitude of the effects is small [41]. Currently, to our knowledge, no such data is published for the TMS. Large education studies and reports regularly point to weaker secondary school performance [42], [43] and Abitur grades among students with low socio-economic status (e.g. mean Abitur grade of 2.27 vs. 2.48 in students transitioning to university with a high vs. low socio-economic background [44]) and a migration background (e.g. mean Abitur grade of 2.5 vs. 2.9 in students with a German vs. Turkish background [45]). Nevertheless, the exact statistical magnitude of these subgroup differences in current Abitur grades for those interested in studying medicine is unclear. Systematic studies and comparisons of subgroup differences in German selection criteria depending on applicants’ ethnicity and socio-economic background are therefore necessary to evaluate the potential of SJTs to increase or decrease access for these groups and to inform decision makers in their selection strategies. 

Since some participants voiced concern that the 5-minute time frame might disadvantage non-native speakers and those with less typing experience, a study of systematic variation of the time limit might shed more light on whether it has the potential to minimize performance differences. An audiovisual response, which seems to further reduce subgroup differences [13], has recently been added to Casper and could be explored in follow-up studies in their potential for a German test version.

German medical schools are called to consider personal characteristics when selecting students [4] and to use selection criteria that indicate their suitability for medical school and the medical profession [46]. It is therefore essential to demonstrate construct and predictive validity. In our study, Casper correlated with non-cognitive selection criteria and cognitive selection criteria in similar magnitude. Thus, it seems that Casper does not merely measure the personal characteristics we aimed to assess but also cognitive characteristics. Therefore, the usefulness of Casper as a meaningful addition to existing selection criteria remains unclear. We could only consider two OSCE stations for a small subsample of study participants. The lack of reliability in a single OSCE station [30] and range restriction in OSCE scores (i.e. students’ OSCE performance ranged between 52.5% and 100% of achievable points) are potentially limiting factors in our analysis. Future research should aim to look at different outcome measures of personal characteristics such as, for example, supervisor and peer ratings or a combination of relevant OSCE stations over the course of medical school [47]. Ideally, these should be compared to the predictive validity of other selection criteria that are currently used in conjunction with cognitive criteria: the completion of a vocational training, as well as work and volunteering experience [8]. 

Finally, from a practical point of view, medical schools need to weigh the costs of a test format like Casper in comparison to alternative selection tools and consider different stakeholders’ perspectives. This study demonstrated that with an average rating time of 46 seconds per response, Casper requires less rater time in comparison to multiple mini-interviews with a station time of five to ten minutes [6] and compared to traditional interviews that are less cost efficient in terms of person hours [48]. Likewise, the estimated costs of a maximum of 95 EUR per applicant (2024) are much lower than 450 EUR per applicant (2014) in the Hamburg multiple mini-interview HAM-Int [7]. However, if costs are covered by test fees, the introduction of Casper would come with an additional financial burden for applicants who already pay to take the TMS (100 EUR in 2024) and HAM-Nat (95 EUR in 2024). A vocational training, on the other hand, provides applicants with the opportunity to learn relevant skills and receive a salary but also requires applicants to invest three years into their training before being able to go to medical school. 

## 5. Conclusions

Positive evaluations by test-takers, good internal consistency, and evidence for discriminant and convergent validity in this study confirm that the test format used in Casper is applicable to a German context. Based on the moderate interrater agreement in our study, the number, background, and training of raters need to be considered and carefully monitored if the test is applied in high-stakes selection. The potential adverse impact on the diversity of students selected by Casper and the current lack of correlation to OSCE performance require potential adjustments to the test and further investigation into the predictive validity of Casper considering a broader range of outcome criteria. It is important to ensure that the test content is relatable to test takers and that it aligns with the goals of German medical education in order to make the test fit for purpose in German medical school selection. In terms of subgroup differences and validity, our current results do not suggest that an open-ended response SJT like Casper is superior to available German SJTs with a closed-ended response format.

## Ethics approval and informed consent

All participants gave their informed consent to data collection, storage and matching of the data. This study as part of the stav research project was approved by the local ethics committee at the Department of Medical Psychology, University Medical Center Hamburg-Eppendorf (LPEK-0042). All data was handled in accordance with European data protection laws (GDPR).

## Acknowledgements

The authors would like to thank Dieter Münch-Harrach for creating the subtitles for the Casper videos. This study would not have been possible without the volunteer raters from the stav teams in Hamburg, Heidelberg, Münster, Saarbrücken, Berlin and Göttingen as well as members from the Eignung & Auswahl Baden-Wuerttemberg network at the Karlsruhe Institute of Technology, Heidelberg University, DHBW Mannheim, University of Education Weingarten and Pforzheim University.

## Funding

This study was conducted as part of the larger stav research project funded by the Federal Ministry of Education and Research, Germany, project number: 01GK1801A-F.

We acknowledge financial support from the Open Access Publication Fund of UKE - Universitätsklinikum Hamburg-Eppendorf.

## Authors’ ORCIDs

- Mirjana Knorr: [0000-0002-0996-9286]
- Ina Mielke: [0000-0003-1764-5553]
- Dorothee Amelung: [0000-0002-9946-9073]
- Mahla Safari: [0000-0003-0976-8094]
- Oana R. Gröne: [0000-0002-6829-5365]
- Simon M. Breil: [0000-0001-5583-3884]
- Alexander MacIntosh: [0000-0002-5094-3774]

## Competing interests

Alexander MacIntosh is a data scientist at Acuity Insights, the company that develops and distributes Casper. The other authors have no competing interests to declare.

## Supplementary Material

Sociodemographic questionnaire of the stav project (2019 version)

CASPer exit survey

Additional tables

## Figures and Tables

**Table 1:**
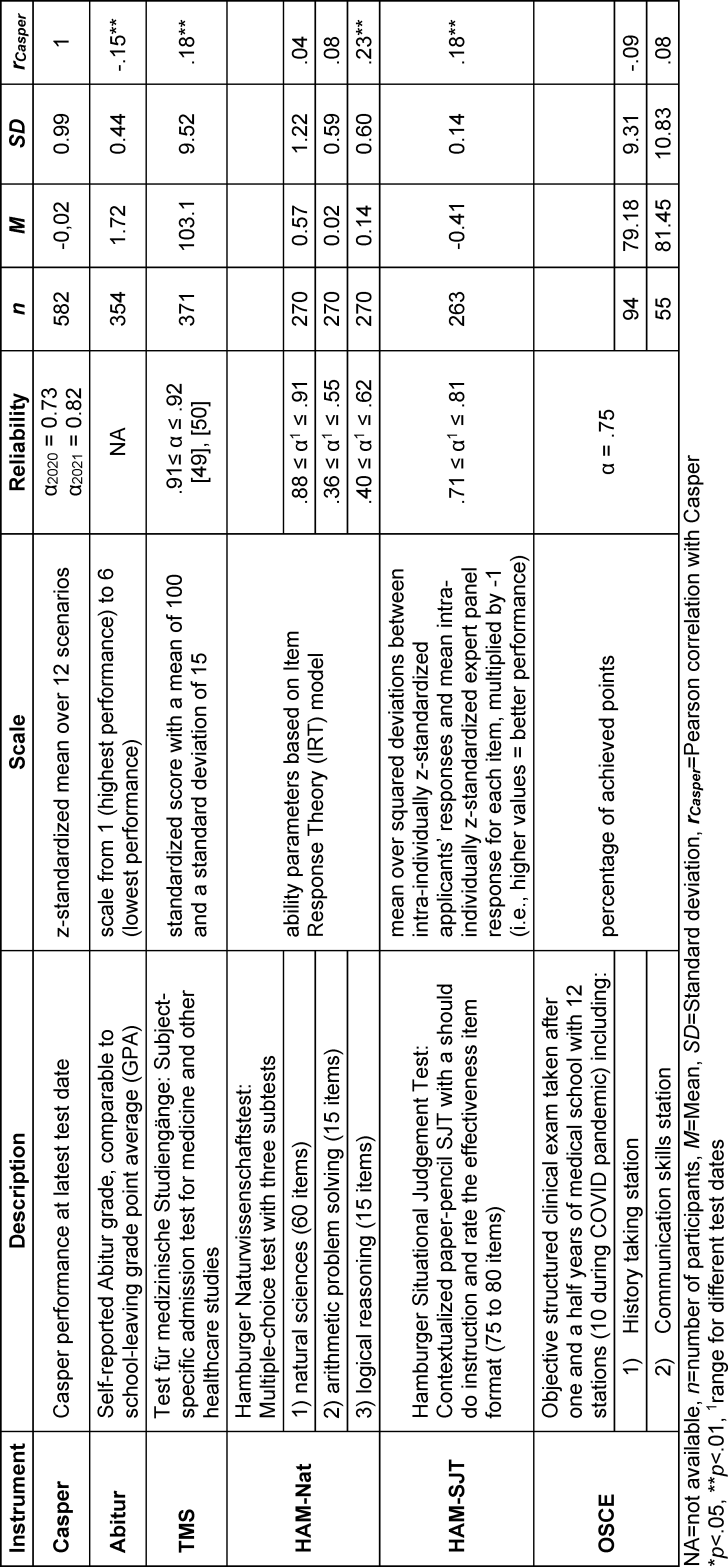
Overview of instruments and their reliability, descriptive statistics in Casper study population, and correlation between Casper score and each of the instruments

**Table 2:**
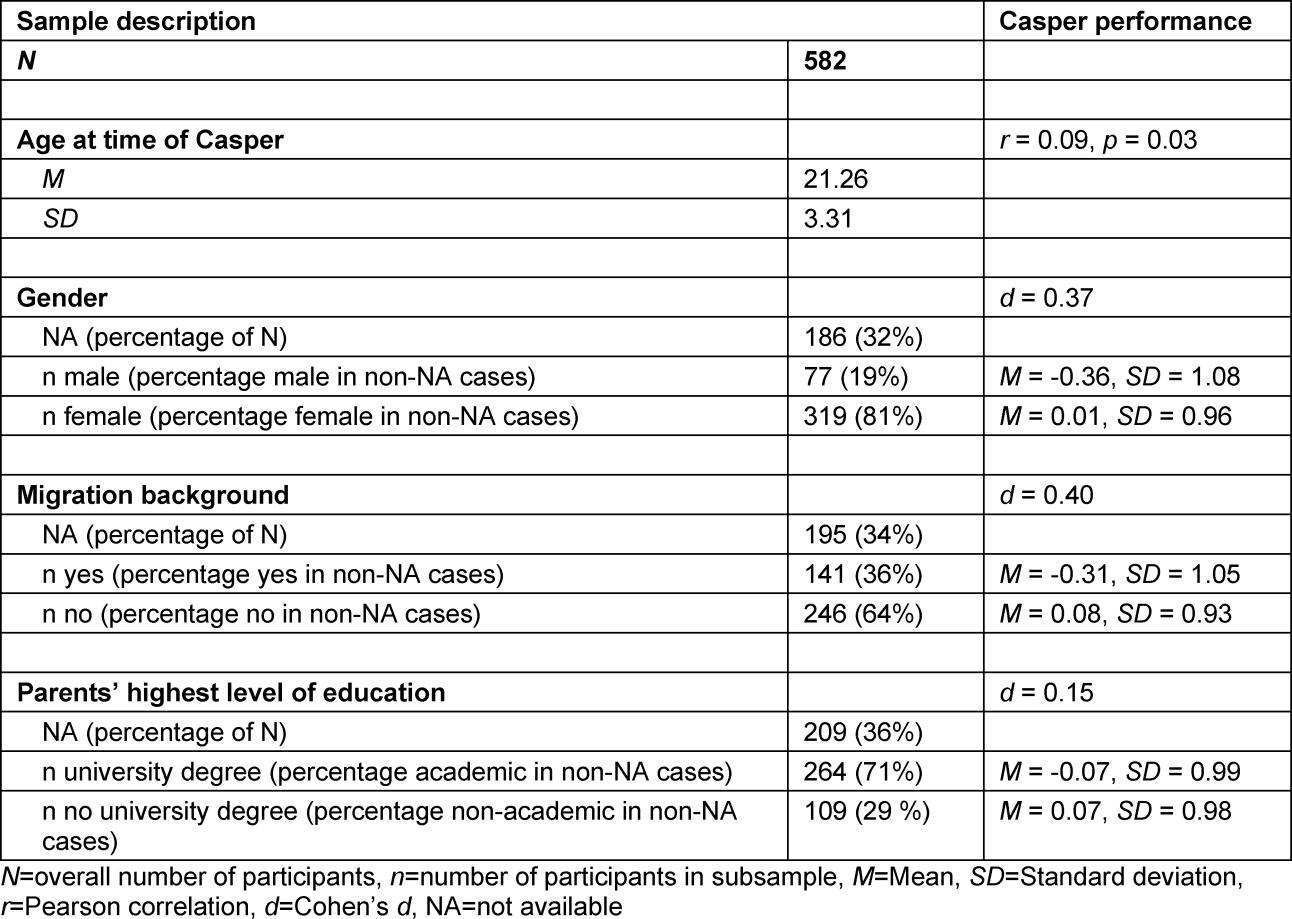
Characteristics of study participants

**Table 3:**
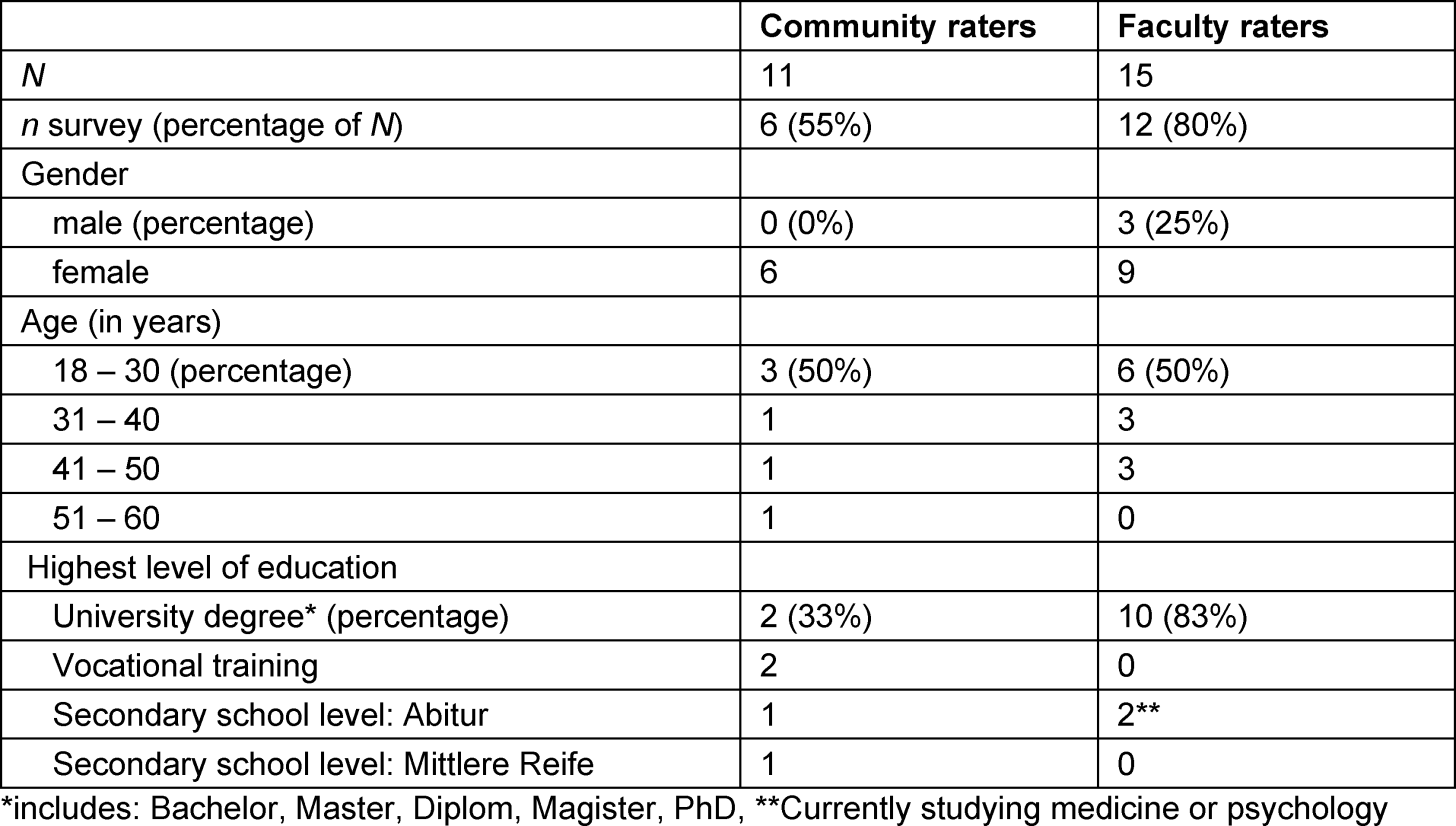
Characteristics of community and faculty raters in 2021

**Table 4:**
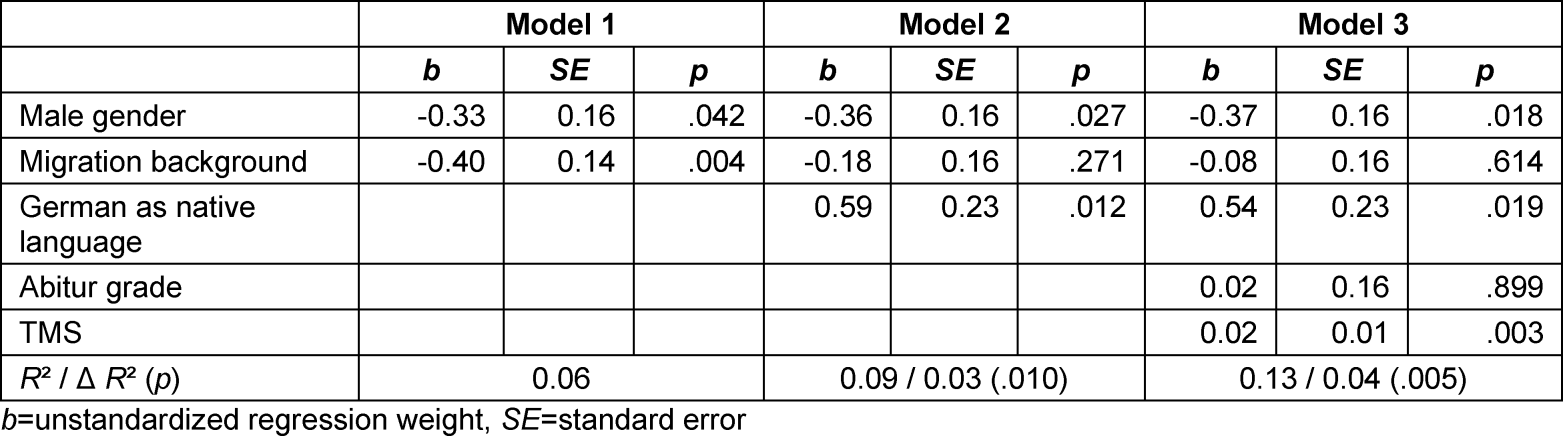
Multiple regression analyses predicting Casper by sociodemographic variables (model 1) controlling for native language (model 2) and cognitive ability (model 3) (n=227)
