# Supplementary material for: Measuring personal characteristics in applicants to German medical schools: Piloting an online Situational Judgement Test with an open-ended response format
Source: GMS J Med Educ. 2024 Jun 17;41(3):Doc30. doi: 10.3205/zma001685 (PMC11310783; doi:10.3205/zma001685)
Supplement: Sociodemographic questionnaire of the stav project (2019 version) [file JME-41-30-s-001.pdf]

Attachment 1: Soziodemografischer Fragebogen des stav-Projektes (Version 2019)/Sociodemographic questionnaire of the stav project (2019 version)

Alle Fragen, die in die Casper-Studie eingeschlossen wurden, sind grün markiert/Questions that were included in the Casper study are highlighted in green.

	**Original German version**	**English translation**
1	Welches Geschlecht haben Sie?<br>• weiblich<br>• männlich	What is your gender?<br>• female<br>• male
2	In welchem Jahr wurden Sie geboren?	In which year were you born?
3	Welche Abiturdurchschnittsnote haben Sie (falls Sie Ihr Ergebnis bereits kennen)?	What is your average Abitur grade (if you already know your result)?
4	Welche Fachhochschulreifenote haben Sie (falls Sie Ihr Ergebnis bereits kennen)?	What is your Fachholschulreife [advanced technical college entrance qualification] grade (if you already know your result)?
5	Welche der folgenden weiterführenden Schultypen haben Sie besucht? (Mehrfachauswahl möglich!)<br>• Gymnasium<br>• Gesamtschule<br>• Schule des 2. Bildungsweges<br>• anderer Schultyp	Which of the following types of secondary school did you attend? (Multiple selection possible!)<br>• secondary/grammar school<br>• comprehensive school<br>• second-chance college<br>• other school type
6	Welchen der nachfolgenden Leistungs- bzw. Schwerpunktkurse hatten Sie belegt? (Mehrfachauswahl möglich!)<br>• Mathematik<br>• Informatik<br>• Physik<br>• Chemie<br>• Biologie<br>• Deutsch<br>• Fremdsprachen<br>• Geschichte<br>• Philosophie/Ethik<br>• Religion<br>• Politik/Wirtschaft<br>• Kunst/Musik<br>• Sport<br>• anderen Leistungs- bzw. Schwerpuntkurs	Which of the following advanced or special subject courses did you take? (Multiple selection possible!)<br>• Mathematics<br>• Computer science<br>• Physics<br>• Chemistry<br>• Biology<br>• German<br>• Foreign Languages<br>• history<br>• Philosophy/Ethics<br>• Religion<br>• Politics/Economics<br>• Art/Music<br>• Sports<br>• Other advanced or special subject course
7	Bitte geben Sie Ihren Familienstand an:<br>• ledig<br>• verheiratet<br>• geschieden<br>• verwitwet	Please state your family status:<br>• single<br>• married<br>• divorced<br>• widowed
8	Wie viele Kinder haben Sie?<br>• kein Kind<br>• 1 Kind<br>• 2 Kinder	How many children do you have?<br>• no child<br>• 1 child<br>• 2 children
9	Ist Deutsch Ihre Muttersprache? (ja/nein)	Is German your mother tongue? (yes/no)
10	Sind Sie in Deutschland geboren? (ja/nein)	Were you born in Germany? (yes/no)
11	Wie lange leben Sie bereits in Deutschland?<br>• ab Beginn des Studiums<br>• weniger als 1 Jahr<br>• 1 bis 3 Jahre<br>• 3 bis 5 Jahre<br>• mehr als 5 Jahre	How long have you been living in Germany?<br>• from the beginning of your studies<br>• less than 1 year<br>• 1 to 3 years<br>• 3 to 5 years<br>• more than 5 years
12	Sind Ihre Eltern in Deutschland geboren?<br>• nein<br>• ein Elternteil<br>• beide Elternteile	Were your parents born in Germany?<br>• no<br>• one parent<br>• both parents

Attachment 1 to Knorr M, Mielke I, Amelung D, Safari M, Gröne OR, Breil SM, MacIntosh A. *Measuring personal characteristics in applicants to German medical schools: Piloting an online Situational Judgement Test with an open-ended response format*. GMS J Med Educ. 2024;41(3):Doc30. DOI: 10.3205/zma001685

13	Bitte geben Sie Ihre Staatsangehörigkeit(en) an: (Mehrfachauswahl möglich!)<br>• Deutsch<br>• sonstige EU-Staatsangehörigkeit<br>• Nicht-EU Staatsangehörigkeit	Please indicate your citizenship(s): (Multiple selection possible!)<br>• German<br>• other EU citizenship<br>• Non-EU citizenship
14	Welchen Abschluss hat Ihre Mutter?<br>• abgeschlossenes Medizinstudium<br>• abgeschlossenes Studium<br>• abgeschlossenes Fachhochschulstudium<br>• Abitur<br>• Meisterprüfung<br>• abgeschlossene Lehre<br>• Realschulabschluss<br>• Hauptschulabschluss<br>• ohne Abschluss<br>• ist mir nicht bekannt	What degree does your mother have?<br>• completed medical studies<br>• completed university studies<br>• completed studies at a university of applied sciences<br>• Abitur<br>• master craftsman's diploma<br>• completed apprenticeship<br>• Realschulabschluss [general certificate of secondary education]<br>• Hauptschulabschluss [lower secondary school leaving certificate]<br>• without qualification<br>• not known to me
15	Welchen Abschluss hat Ihr Vater?<br>• abgeschlossenes Medizinstudium<br>• abgeschlossenes Studium<br>• abgeschlossenes Fachhochschulstudium<br>• Abitur<br>• Meisterprüfung<br>• abgeschlossene Lehre<br>• Realschulabschluss<br>• Hauptschulabschluss<br>• ohne Abschluss<br>• ist mir nicht bekannt	What degree does your father have?<br>• completed medical studies<br>• completed university studies<br>• completed studies at a university of applied sciences<br>• Abitur<br>• master craftsman's diploma<br>• completed apprenticeship<br>• Realschulabschluss [general certificate of secondary education]<br>• Hauptschulabschluss [lower secondary school leaving certificate]<br>• without qualification<br>• not known to me
16	Sind ihre Eltern im Gesundheitswesen beschäftigt?<br>• beide Elternteile<br>• ein Elternteil<br>• nein	Are your parents employed in the health sector?<br>• both parents<br>• one parent<br>• no
17	Waren Sie mindestens ein halbes Jahr im Gesundheits- und Sozialwesen beschäftigt? (ja/nein)	Have you been employed in the health and social care sector for at least half a year? (yes/no)
18	Haben Sie bereits eine Berufsausbildung abgeschlossen?<br>• nein<br>• ja, im nicht-medizinischen Bereich<br>• im medizinischen Bereich<br>• ja, im zahnmedizinischen Bereich	Have you already completed vocational training?<br>• no<br>• yes, in the non-medical field<br>• in the medical field<br>• yes, in the dental field
19	Haben Sie bereits eine Berufsausbildung angefangen und nicht beendet?<br>• nein<br>• ja, im nicht-medizinischen Bereich<br>• im medizinischen Bereich<br>• ja, im zahnmedizinischen Bereich	Have you already started vocational training and not completed it?<br>• no<br>• yes, in the non-medical field<br>• in the medical field<br>• yes, in the dental field
20	Haben Sie bereits ein anderes Studium abgeschlossen? (ja/nein)	Have you already completed another degree? (yes/no)
21	Haben Sie bereits ein anderes Studium angefangen und nicht beendet? (ja/nein)	Have you already started another degree programme and not finished it? (yes/no)
