# Supplementary material for: Measuring personal characteristics in applicants to German medical schools: Piloting an online Situational Judgement Test with an open-ended response format
Source: GMS J Med Educ. 2024 Jun 17;41(3):Doc30. doi: 10.3205/zma001685 (PMC11310783; doi:10.3205/zma001685)
Supplement: CASPer exit survey [file JME-41-30-s-002.pdf]

Attachment 2: Evaluationsfragebogen zu CASPer/CASPer exit survey

	German	English	*n*	*min*	*max*	*M*	*SD*
1	Wie bewerten Sie CASPer insgesamt?<br>(1 = extrem negativ,<br>10 = extrem positiv)	Overall how do you rate CASPer?<br>(1 = extremely negatively,<br>10 = extremely positively)	368	2	10	7.55	1.64
2	Auf einer Skala von 1 bis 7, wie zufrieden sind Sie insgesamt mit der CASPer-Testerfahrung?<br>(1 = extrem unzufrieden,<br>7 = extrem zufrieden)	On a scale from 1 to 7, how satisfied are you with the overall CASPer test experience?<br>(1 = extremely dissatisfied,<br>7 = extremely satisfied)	367	1	7	5.40	1.18
3	Wie hätten wir die CASPer-Testerfahrung für Sie noch besser machen können?<br>(Offene Textantwort)	How could we have made the overall CASPer test experience better for you?<br>(Open text response)					
4	Auf einer Skala von 1 bis 7, wie fair ist CASPer aus Ihrer Sicht zu allen Teilnehmer*innen?<br>(1 = gar nicht fair,<br>7 = extrem fair)	On a scale from 1 to 7, how fair do you think CASPer is to all students?<br>(1 = not fair at all,<br>7 = extremely fair)	354	2	7	5.24	1.26
5	Auf einer Skala von 1 bis 7, wie gut hat CASPer Ihnen aus Ihrer Sicht die Möglichkeit geboten, Ihre Stärken im Vergleich zu anderen Teilnehmer*innen zu zeigen.<br>(1 = gar nicht gut,<br>7 = extrem gut)	On a scale from 1 to 7, how well do you think CASPer allowed you to demonstrate your strengths relative to other applicants?<br>(1 = not well at all,<br>7 = extremely well)	348	1	7	4.44	1.47
6	Auf einer Skala von 1 bis 7, wie effektiv finden Sie CASPer als Instrument für die Beurteilung der persönlichen und professionellen Eigenschaften einer Person für den Beruf?<br>(1 = gar nicht effektiv,<br>7 = extrem effektiv)	On a scale from 1 to 7, how effective do you think CASPer is as a tool for evaluating one's personal and professional characteristics for the profession?<br>(1 = not effective at all,<br>7 = extremely effective)	355	1	7	4.72	1.46
7	Auf einer Skala von 1 bis 7, wie anstrengend war der CASPer im Vergleich zu anderen Prüfungen?<br>(1 = viel weniger anstrengend,<br>7 = sehr viel anstrengender)	On a scale from 1 to 7, compared to other exams, how stressful was the CASPer test?<br>(1 = much less stressful,<br>7 = much more stressful)	359	1	7	3.24	1.50
8	Auf einer Skala von 1 bis 7, wie leicht oder schwer fanden Sie den CASPer?<br>(1 = extrem leicht,<br>7 = sehr schwer)	On a scale from 1 to 7, how easy or difficult did you find the CASPer test?<br>(1 = extremely easy,<br>7 = extremely difficult)	356	1	7	4.08	1.21
9	Auf einer Skala von 1 bis 7, wie würden Sie Ihre eigene Leistung im CASPer einschätzen?<br>(1 = gar nicht gut,<br>7 = extrem gut)	On a scale from 1 to 7, how would you evaluate your performance on the CASPer test?<br>(1 = not good at all,<br>7 = extremely good)	355	1	7	4.53	0.96

Attachment 2 to Knorr M, Mielke I, Amelung D, Safari M, Gröne OR, Breil SM, MacIntosh A. *Measuring personal characteristics in applicants to German medical schools: Piloting an online Situational Judgement Test with an open-ended response format*. GMS J Med Educ. 2024;41(3):Doc30. DOI: 10.3205/zma001685

10	Auf einer Skala von 1 bis 7, was glauben Sie, wie sehr sich Ihre eigene Leistung in den verschiedenen Szenarien des CASPer unterscheidet?<br>(1 = gar keine Unterschiede,<br>7 = extreme Unterschiede)	On a scale from 1 to 7, to what extend do you believe does your performance differ between the scenarios of the CASPer test?<br>(1 = no differences at all,<br>7 = extreme differences)	351	2	7	3.60	1.08
11	Auf einer Skala von 1 bis 7, wird es wahrscheinlicher oder weniger wahrscheinlich, dass Sie sich bei einer Fakultät bewerben, wenn der CASPer dort eine Voraussetzung ist?<br>(1 = viel weniger wahrscheinlich,<br>7 = viel wahrscheinlicher)	On a scale from 1 to 7, does having the CASPer test as a requirement make you more likely or less likely to apply to a school?<br>(1 = much less likely,<br>7 = much more likely)	352	1	7	4.90	1.59
12	Welche Strategien haben Sie genutzt, um sich auf den CASPer vorzubereiten. Wählen Sie alle zutreffenden Optionen aus.<br>1. Durchsehen des Materials für Bewerber*innen auf der Take CASPer Internetseite (z.B. FAQs, Blogs, Webinar)<br>2. Durchführung des 12-teiligen Übungstests in Ihrem CASPer Account<br>3. Teilnahme an einem CASPer Vorbereitungskurs eines Drittanbieters<br>4. Übung potentieller CASPer-Fragen auf Basis der CASPer-Kompetenzen<br>5. Übung von Antworten mit technischer Hilfe<br>6. Übung von Antworten ohne technische Hilfe<br>7. Ich habe mich nicht auf den CASPer vorbereitet.	What strategies did you use to prepare for the CASPer test? Check all that apply.<br>1. Reviewed the applicant resources on the Take CASPer website (e.g. FAQs, blogs, webinar)<br>2. Completed the 12-section practice test in your CASPer account<br>3. Participated in a third-party CASPer test preparation course<br>4. Studied potential CASPer questions based on the competencies<br>5. Rehearsed responses with technology<br>6. Rehearsed responses without technology<br>7. Did not prepare for the CASPer test					
13	Welche anderen Strategien haben Sie für die Vorbereitung auf CASPer genutzt, die in der Liste nicht aufgeführt waren?<br>(Offene Textantwort)	Which other strategies did you used to prepare for CASPer that are not listed above?<br>(Open text response)					
14	Hatten Sie Kontakt zum Altus Kunden-Support während des CASPer?<br>Ja<br>Nein	Did you interact with customer support during your CASPer test?<br>Yes<br>No					
15	Auf einer Skala von 1 bis 7, wie zufrieden waren Sie mit dem Kunden-Support, den Sie erhalten haben?<br>(1 = extrem unzufrieden,<br>7 = extrem zufrieden)	On a scale from 1 to 7, how satisfied are you with the customer support you received?<br>(1 = extremely dissatisfied,<br>7 = extremely satisfied)	117	2	7	6.44	1.02
16	Wie hätte unser Team Sie noch besser unterstützen können?<br>(Offene Textantwort)	How could our team have supported you better?<br>(Opened text response)					

17	Auf einer Skala von 1 bis 7, in welchem Ausmaß hat die CASPer Internetseite Ihnen die Hilfsmittel geboten, die Sie für ein reibungsloses CASPer-Erlebnis von dem Zeitpunkt der Account-Erstellung bis zum Zeitpunkt der Ergebnisübermittlung gebraucht hätten.<br>(1 = überhaupt nicht gut,<br>7 = extrem gut)	On a scale from 1 to 7, to what extent did the CASPer website provide you with the resources you needed to have a smooth experience with CASPer from time of account creation to time of results delivery<br>(1 = not well at all,<br>7 = extremely well)	316	1	7	5.47	1.31
18	Hatten Sie irgendwelche technischen Probleme während Ihres Tests?	Did you experience any technical issues during your test?					
19	Welche Art von Problemen hatten Sie? Wählen Sie alle zutreffenden Optionen aus.<br>1. Webcam hat nicht funktioniert<br>2. Video hat zu lange geladen oder geruckelt<br>3. Video ist stehen geblieben<br>4. Video hatte Tonprobleme<br>5. Ich konnte nicht in das Antwortfeld tippen.<br>6. Ich wurde nicht zur nächsten Seite weitergeleitet, nachdem ich auf Abschicken geklickt hatte.<br>7. Seite hat eine Fehlermeldung angezeigt<br>8. Seite ist abgelaufen/wurde nicht geladen<br>9. Internetverbindung wurde unterbrochen<br>10. Andere – Bitte erläutern Sie	What kind of issues did you experience? Check all that apply.<br>1. Webcam did not work<br>2. Video buffered for too long or stuttered<br>3. Video froze<br>4. Video had audio problems<br>5. Could not type into answer field<br>6. Page did not progress after timer expired<br>7. Page did not progress after I clicked submit<br>8. Page generated error message<br>9. Page expired/did not load<br>10. Internet disconnected<br>Other - Please explain					
20	Bitte erläutern Sie das Problem / die Probleme genauer:	Please explain the issue(s) in detail:					
21	Haben Sie irgendwelche Anmerkungen zu einem CASPer Szenario? Fanden Sie zum Beispiel ein Szenario besonders schwierig oder interessant? Bitte erläutern Sie dies genauer.	Do you have any comments about a CASPer scenario? For example, did you find any of them to be particularly difficult or interesting? Please describe in detail.					
22	Gab es irgendwelche Szenarien, die Sie nicht verstehen oder mit denen Sie sich nicht identifizieren konnten? Bitte erläutern Sie dies genauer.	Were there any scenarios that you were unable to understand or relate to? Please describe in detail.					
23	Haben Sie irgendeine andere Rückmeldung, die Sie uns gerne mitteilen wollen?	Do you have any other feedback you would like to share with us?					

Common themes in open text feedback (excluding reports of technical issues) / Allgemeine Themen im offenen Text-Kommentaren (ohne Berichte über technische Probleme)

Comments related to	Common topics (frequency)	Kommentare zu	Allgemeine Themen (Häufigkeit)
Test experience	• Response time was too short, test depends too much on individual typing speed (n = 124) vs. time pressure was adequate (n=4)<br>• Test material should be in one language only, German videos would have been better (n= 25)<br>• More breaks between scenarios, more time to think about a scenario (n = 21) vs. enough time (n = 1)<br>• allow bullet points, audio/video response or special accommodations for disadvantaged applicants (n = 16)<br>• Overall test experience was good/fun (n = 13)<br>• would have preferred (additional) multiple choice questions (n = 7) vs. preferred open response format (n = 5)<br>• Allow possibility to rewind or replay video scenario (n = 6)<br>• preferred video scenarios (n = 5) vs. preferred text scenarios (n = 1)<br>• Questions should be presented together with scenario, before the start of the response time (n = 5)<br>• overall test length too long (n =4)<br>• Would prefer on-side testing (n = 2) vs. online test (n = 1)	Testerfahrung	• Antwortzeit war zu kurz, Test hängt zu sehr von der individuellen Tippgeschwindigkeit ab (n= 124) vs. Zeitdruck war angemessen (n=4)<br>• Testmaterial sollte nur in einer Sprache sein, deutsche Videos wären besser gewesen (n= 25)<br>• Mehr Pausen zwischen den Szenarien, mehr Zeit zum Nachdenken über ein Szenario (n = 21) vs. genug Zeit (n = 1)<br>• Stichpunkte, Audio-/Videoantworten oder besondere Vorkehrungen für benachteiligte Bewerber zulassen (n = 16)<br>• Das Testerlebnis war insgesamt gut/hat Spaß gemacht (n = 13)<br>• hätte (zusätzliche) Multiple-Choice-Fragen bevorzugt (n = 7) vs. bevorzugt offenes Antwortformat (n = 5)<br>• Möglichkeit zum Zurückspulen oder Wiederholen des Videoszenarios (n = 6)<br>• Bevorzugt Videoszenarien (n = 5) vs. bevorzugt Textszenarien (n = 1)<br>• Die Fragen sollten zusammen mit dem Szenario präsentiert werden, bevor die Antwortzeit beginnt (n = 5)<br>• Gesamtlänge des Tests zu lang (n = 4)<br>• würde Vor-Ort-Tests bevorzugen (n = 2) vs. Online-Test (n = 1)
Test concept	• Overall test concept is interesting/good, would be a good addition to medical school selection (n = 19) vs. was not convincing (n = 2)<br>• Scoring and what is expected in a good response is unclear (n = 11)<br>• Should be more specific to medical context / based on job/study-relevant scenarios (n = 10)<br>• Would prefer personal interview (n = 9)<br>• Written response is not necessarily indicative of actual behaviour (n = 9)<br>• quality of videos/actors praised (n = 3) vs. criticized (n = 4)	Testkonzept	• Gesamtkonzept des Tests ist interessant/gut, wäre eine gute Ergänzung für die Auswahl von Medizinstudierenden (n = 19) vs. war nicht überzeugend (n = 2)<br>• Punktevergabe und Erwartungen an eine gute Antwort sind unklar (n = 11)<br>• Sollte spezifischer auf den medizinischen Kontext ausgerichtet sein / auf berufs-/studienrelevanten Szenarien basieren (n = 10)<br>• Würde ein persönliches Gespräch bevorzugen (n = 9)<br>• Schriftliche Antwort ist nicht unbedingt ein Indikator für das tatsächliche Verhalten (n = 9)<br>• Qualität der Videos/Schauspieler gelobt (n = 3) vs. kritisiert (n = 4)

Test content	Scenario perceived as difficult if:<br>• it required participant to think of a past experience: too little time to think of a scenario, question too private (n = 75)<br>• the situation was unfamiliar (e.g. working for a company, owning a car) (n = 26)<br>• it lacked of more detailed background information (n = 12)<br>• if was perceived as Clichés or too trivial (n = 4)<br>• it felt constructed, abstract or very unlikely (n = 6)<br>• legal knowledge was required (n = 4)<br>• it was a dilemma between one's own interests and the interest of others (n = 3)<br>• specific for a North American context (n = 2)<br>• it was a private context (e.g. with family, friends) (n = 1)<br>Scenarios perceived as interesting<br>Overall<br>• Scenarios were interesting, comprehensible (n = 24)<br>• Questions to scenarios could be more precise (n = 17)<br>• Scenarios were similar, responses felt similar between scenarios (n = 11)<br>• Scenarios could have been more challenging (n = 5)	Testinhalte	Ein Szenario wurde als schwierig empfunden, wenn:<br>• Teilnehmer*in an eine frühere Erfahrung zurückdenken musste: zu wenig Zeit, um an ein Szenario zu denken, zu private Frage (n = 75)<br>• die Situation ungewohnt war (z. B. Arbeit in einem Unternehmen, Besitz eines Autos) (n = 26)<br>• detailliertere Hintergrundinformationen fehlten (n = 12)<br>• die Situation als klischeehaft oder zu trivial empfunden wurde (n = 4)<br>• es als konstruiert, abstrakt oder sehr unwahrscheinlich empfunden wurde (n = 6)<br>• juristische Kenntnisse erforderlich waren (n = 4)<br>• es ein Dilemma zwischen den eigenen Interessen und den Interessen anderer war (n = 3)<br>• es spezifisch für einen nordamerikanischen Kontext war (n = 2)<br>• es sich um einen privaten Kontext handelte (z. B. mit Familie, Freunden) (n = 1)<br>Als interessant empfundene Szenarien<br>Insgesamt<br>• Szenarien waren interessant, verständlich (n = 24)<br>• Die Fragen zu den Szenarien könnten präziser sein (n = 17)<br>• Szenarien waren ähnlich, Antworten fühlten sich zwischen den Szenarien ähnlich an (n = 11)<br>• Die Szenarien hätten anspruchsvoller sein können (n = 5)
