# Supplementary material for: Measuring personal characteristics in applicants to German medical schools: Piloting an online Situational Judgement Test with an open-ended response format
Source: GMS J Med Educ. 2024 Jun 17;41(3):Doc30. doi: 10.3205/zma001685 (PMC11310783; doi:10.3205/zma001685)
Supplement: Additional tables [file JME-41-30-s-003.pdf]

Attachment 3: Additional tables

Table 1A: Sociodemographic characteristics in each study cohort

	**2020**	**2021**		**Subsample: 2020 and 2021**
**N**	**391**	**211**		**20**
Test date 1	213	128		
Test date 2	93	83		
Test date 3	85	-		
**Age (at time of Casper)**				
M	21.32	21.46		25.6
SD	3.01	4.22		6.04
Δ2020-2021	*t*(327.63) = -0.42, *p* = 0.67, *d* = -0.04			
**Gender**				
NA (percentage of N)	61 (16%)	133 (63%)		8 (40%)
n male (percentage male in non-NA cases)	63 (19%)	19 (24%)		5 (42%)
n female	267	59		7
**Migration background**				
NA (percentage of N)	67 (17%)	136 (66%)		8 (40%)
n yes (percentage yes in non-NA cases)	116 (36%)	31 (41%)		6 (50%)
n no	208	44		6
**Parents' highest level of education**				
NA (percentage of N)	77 (20%)	140 (66%)		8 (40%)
n academic (percentage academic in non-NA cases)	226 (72%)	48 (68%)		10 (83%)
n no academic background	88	23		2

Participants needed to provide their birth date during the registration for Casper. Therefore, information about age was available for the whole sample. There was no significant difference in age between the two samples.

Other socio-demographic characteristics were assessed in a voluntary questionnaire. For the 2021 sample sociodemographic data is missing for more than 60% - possibly because many applicants in this sample (TMS participants) were not invited to fill-out the sociodemographic questionnaire.

Attachment 3 to Knorr M, Mielke I, Amelung D, Safari M, Gröne OR, Breil SM, MacIntosh A. *Measuring personal characteristics in applicants to German medical schools: Piloting an online Situational Judgement Test with an open-ended response format*. GMS J Med Educ. 2024;41(3):Doc30. DOI: 10.3205/zma001685

Table 2A: Descriptive statistics of predictor and outcome variables for each study cohort

	2020 study				2021 study			
	n	% of overall sample	mean	sd	n	% of overall sample	mean	sd
**Casper20**	391	100%	0.00	0.99	20	9%	0.28	0.98
**Casper21**	20	5%	0.25	1.25	211	100%	-0.01	0.99
**Casper_last**	391	100%	-0.01	1.01	211	100%	-0.01	0.99
**Abitur**	300	77%	1.72	0.43	66	31%	1.74	0.48
**TMS**	234	60%	102.80	9.67	142	67%	103.63	9.30
**HAM-Nat SC**	209	53%	0.64	1.19	66	31%	0.38	1.28
**HAM-Nat AP**	209	53%	0.04	0.59	66	31%	-0.04	0.59
**HAM-Nat RS**	209	53%	0.17	0.58	66	31%	0.04	0.65
**HAM-SJT**	209	53%	-0.43	0.15	59	28%	-0.35	0.11
**MMI**	21	5%	3.37	0.39	1	0%	3.72	
**Cognitive study success**	67	17%	82.09	6.91	22	10%	82.90	8.04
**OSCE overall performance**	82	21%	85.95	4.41	25	12%	85.45	4.46
**OSCE station patient history**	82	21%	79.24	9.47	25	12%	78.90	8.54
**OSCE station communication skills**	46	12%	81.85	11.37	16	8%	80.00	7.75

SC=natural science subtest, AP=arithmetic problem solving subtest, RS=logical reasoning subtest

For most of the variables, unpaired Welch t-Tests and Mann-Whitney-U-Tests did not reveal any significant differences between the two samples. HAM-SJT values significantly differed between the 2020 and 2021 study ($W=3773.5$, $p<.001$).

For those who participated in the study in both years (n=20), the rank correlation of Casper performance between years was small ($\rho=0.29$, $p=0.22$).

Table 3A: Descriptive statistics and Pearson correlations between all study variables

	N	***M***	***SD***	**1**	**2**	**3**	**4**	**5**	**6**	**7**	**8**
1. Casper	582	-0,02	0.99								
2. Abitur	354	1.72	0.44	-.15**							
3. TMS	371	103.1	9.52	.18**	-.35** (*n* = 230)						
4. HAM-Nat SC	270	0.57	1.22	.04	-.36** (*n* = 242)	.19* (*n* = 168)					
5. HAM-Nat AP	270	0.02	0.59	.08	-.16* (*n* = 242)	.34** (*n* = 168)	.27** (*n* = 270)				
6. HAM-Nat RS	270	0.14	0.6	.23**	-.15* (*n* = 242)	.36** (*n* = 168)	.23** (*n* = 270)	.43** (*n* = 270)			
7. HAM-SJT	263	-0.41	0.14	.18**	-.07 (*n* = 239)	.05 (*n* = 167)	-.01 (*n* = 263)	.08 (*n* = 263)	.07 (*n* = 263)		
8. OSCE "history taking"	94	79.18	9.31	-.09	-.25 (*n* = 38)	.09 (*n* = 21)	.36 (*n* = 18)	-.15 (*n* = 18)	-.02 (*n* = 18)	-.17 (*n* = 18)	
9. OSCE "communication"	55	81.45	10.83	.08		.30 (*n* = 8)					.17 (*n* = 55)

*p<.05, **p<.01; *N*=sample size within Casper participant population, *n*=size of subsample, *M*=mean, *SD*=standard deviation, SC=natural science subtest, AP=arithmetic problem solving subtest, RS=logical reasoning subtest
